# Supplementary material for: Expansion of the Group B Streptococcus serotype repertoire via gene acquisition from other streptococcal species
Source: Microbiol Spectr. 2025 Oct 27;13(12):e01227-25. doi: 10.1128/spectrum.01227-25 (PMC12671096; doi:10.1128/spectrum.01227-25)
Supplement: Fig. S1 — ELISA data of wild-type VIII strains. [file spectrum.01227-25-s0001.docx]

**Supplemental Figure 1.** Whole cell enzyme-linked immunosorbent assay (ELISA) using serotype VIII-specific primary antibody to assess relative levels of total capsule. Dots represent technical replicates (n = 8), and the red bar represents the median. Strain numbers and descriptions located in Table S1**.**
