# Supplementary material for: Expansion of the Group B Streptococcus serotype repertoire via gene acquisition from other streptococcal species
Source: Microbiol Spectr. 2025 Oct 27;13(12):e01227-25. doi: 10.1128/spectrum.01227-25 (PMC12671096; doi:10.1128/spectrum.01227-25)
Supplement: Supplemental tables — Tables S1 and S3. [file spectrum.01227-25-s0002.docx]

**Supplemental Table 1.** Wild-type, serotype VIII strains of GBS used in this study

| AR Strain # | Description | CDC strain # |
| --- | --- | --- |
| AR967 | Serotype VIII | CDC strain 5030-08 |
| AR977 | Serotype VIII used as the genetic background for all mutagenesis work in this study | CDC strain 2013226269 |
| AR1187 | Serotype VIII | CDC strain 20140487 |
| AR1188 | Serotype VIII | CDC strain 20150287 |
| AR1189 | Serotype VIII | CDC strain 2014207299 |
| AR2204 | Serotype VIII, ST 42 | CDC strain 20162186 |
| AR2205 | Serotype VIII, ST 42 | CDC strain 20162221 |
| AR2206 | Serotype VIII, ST 2 | CDC strain 20162079 |
| AR2207 | Serotype VIII, ST 2 | CDC strain 20162571 |
| AR2208 | Serotype VIII | CDC strain 20163358 |
| AR2209 | Serotype VIII, ST 42 | CDC strain 20164226 |
| AR2210 | Serotype VIII | CDC strain 20164682 |
| AR2211 | Serotype VIII, ST 2 | CDC strain 20166504 |
| AR2212 | Serotype VIII, ST 42 | CDC strain 20166744 |
| AR2213 | Serotype VIII, ST 42 | CDC strain 20170303 |
| AR2214 | Serotype VIII, ST 42 | CDC strain 20171014 |
| AR2215 | Serotype VIII, ST 42 | CDC strain 20173348 |
| AR2216 | Serotype VIII, ST 1 | CDC strain 20174072 |
| AR2217 | Serotype VIII, ST 42 | CDC strain 20176038 |
| AR2218 | Serotype VIII, ST 42 | CDC strain 20180064 |
| AR2219 | Serotype VIII, ST 42 | CDC strain 20180184 |
| AR2220 | Serotype VIII, ST 2 | CDC strain 20180206 |

**Supplemental Table 3.** Primers and gBlocks (IDT) used in this study

| FR824043_cpsF_fwd  FR824043_cpsF_rev | Suis_cps34F_fwd  Suis_cps34F_rev | cpsR_fwd  cpsR_rev | cps8E_KOcheck_fwd  cps8E_KOcheck_rev | Downstream_cps8E_fwd  Downstream_cps8E_rev | Upstream_cps8E_fwd  Upstream_cps8E_rev | pOri23_BamHI_fwd  pOri23_PstI_rev | | cps8R_fwd  cps8R_rev | cpsR_check_fwd  cpsR_check_rev | cpsRKO_junction_rev | pMBSacB_check_fwd  pMBSacB_check_rev | Downstream_cpsR_fwd  Downstream_cps_rev | Upstream_cpsR_fwd  Upstream_cpsR_rev | Primer / gBlock |  |
| --- | --- | --- | --- | --- | --- | --- | --- | --- | --- | --- | --- | --- | --- | --- | --- |
| tatgaatgacaatgatgttgATGAAAAGTGTCTATATTATAGGTTC  aatcgataagcttggctgcaTTATACTTTTTCCTCAAACAATC | tatgaatgacaatgatgttgATGACTAAATCTGTATACATTATTGG  aatcgataagcttggctgcaTTATCCTTTAAATAACTGCTCATAC | TGTGGTAGGTACTCGTCCTC  GGAACCTCCTAAAACAATCCCCA | CGGGTTTATTGTTGGTGCAGG  GTCCCTCCAACTTCATGACCA | agaataagaaTGGAATTATTTACTCTCTTAAATTTTATAAAATTTAAG  tggagctccaccgcggtggcCAAGATCTCTTTTTGATTTAGATTTC | attgggtaccgggcccccccTGTTAATACTGAAGAAGATATTGAAAAG  aataattccaTTCTTATTCTAAAACCTATCTTTTTC | AGCCCTGACAACCCTTGTTC  TACCGCCTTTGAGTGAGCTG | | tatgaatgacaatgatgttgATGAGAAGGACAGTATATATTATTG  aatcgataagcttggctgcaTTATTGATTTTTAGAGTCATAATGTTTAC | CGCAGAAACGTGTTGGAAAGA  TCCTCCAAAAGCAAGCTGTG | ACCGTTAAAGTTGCCAAACCAG | TGCAAGGCGATTAAGTTGGGTAA  GCGGATAACAATTTCACACAGGAAA | tggaggagttATGACTCTAAAAATCAATAATTTATTTTTTTC  tggagctccaccgcggtggcCCCTTGAACTCTATAAGTATAG | attgggtaccgggcccccccCAGCACGATATTTTAAACTACTTG  ttagagtcatAACTCCTCCATTCTCTTAAATTTTATAAAATTTAAG | Sequence (5’to 3’) |  |
| Amplifies *cpsF* from gBlock for Gibson assembly into pOri23 digested with BamHI and PstI | Amplifies *cpsF* from gene block for Gibson assembly into pOri23 digested with BamHI and PstI | Binds wild-type VIII gDNA outside of *cpsR* for confirming genotype of colonies recovered from murine co-colonization experiments | Binds wild-type VIII gDNA outside of *ΔcpsE* mutagenesis cassette for confirmation of deletion / revertant genotype | Amplified downstream homology arm for *ΔcpsE* mutagenesis cassette to be Gibson assembled into pMBSacB digested with XhoI and NotI | Amplifies upstream homology arm for *ΔcpsE* mutagenesis cassette to be Gibson assembled into pMBSacB digested with XhoI and NotI | Binds pOri23 outside of BamHI and PstI cut sites for confirmation of complemented gene insertion | | Amplifies *cpsR* for Gibson assembly into pOri23 digested with BamHI and PstI | Binds wild-type VIII gDNA outside of *ΔcpsR* mutagenesis cassette for confirmation of deletion / revertant genotype | Binds *ΔcpsR* mutagenesis cassette internally for sequencing junction between up and downstream homology arms | Binds pMBSacB outside of XhoI and NotI cut sites for confirmation of mutagenesis cassette insertion | Amplifies downstream homology arm for *ΔcpsR* mutagenesis cassette to be Gibson assembled into pMBSacB digested with XhoI and NotI | Amplifies upstream homology arm for *ΔcpsR* mutagenesis cassette to be Gibson assembled into pMBSacB digested with XhoI and NotI | Description |  |
| FR824043_cpsF_gBlock | | | | | | | Suis_cps34F_gBlock | | | | | | | | Primer / gBlock |
| ATGAAAAGTGTCTATATTATAGGTTCAAAAGGAATACCAGCGAATTATGGTGGGTTTGAAACCTTTGTAGAAAAACTAACTGAAAATCAAAAAGATAAAAATATCAAATATTATGTGGCATGTATGCGAGAAAACTCTGCTAAATCAAATATCACAGATGATATTTTTGAACATAATGGAGCAACTTGTTTTAATATTGATGTGCCAAATATTGGTCCTGCACGAGCAATTGCCTATGACATCGCTGCTCTAAGCAGGTCGATTGAAATCGCAAAAGAAAATGGTGATGTTAATCCAATTTTTTATGTTTTAGCTTGTCGCATCGGACCATTCATTAATCATTTTAAAAAGCAAATCCACGCTCTTGGTGGTCAATTATTTGTTAATCCAGATGGACATGAATGGATGCGTCAAAAATGGAGTGCCCCTGTTCGTCGTTATTGGAAAGTTTCTGAGTCATTGATGGTAAAACATGCTGATTTATTGGTATGTGATAGTAAGAATATTGAAAAATATATCCAAGATGACTATAAAAAATATTCACCAAAGACAACTTACATCGCTTATGGGACAGAGCTTGAAAAATCTAGCCTTTCTTCAAAAGACCGTGTCGTACGTGAATGGTTTTCAGAAAAGGAAGTTTCAGAAAATAATTATTACCTTGTTGTTGGACGATTCGTTCCAGAAAATAACTATGAGGCAATGCTTCGTGAGTTTATGAAGTCAAATACAAAAAAAGATTTTGTATTGGTTACTAATGTTGAACAAAATGCCTTTTACGAAAAATTGAAGAAAGAAACAGGTTTTGACAAAGACTCTCGCATCAAATTTGTAGGAACTGTTTATAATCAAGAACTTCTTAAATATATTCGTGAAAATGCATATGCTTACTTCCACGGTCATGAAGTTGGTGGAACAAATCCATCACTATTAGAAGCATTATCTTCAACTAAGTTAAACCTCTTGCTTAATGTTGGATTTAACAAAGAAGTTGGTGGAGATGGAGCGATTTATTGGGATAAAGATAATCTTCATAAGGTTATTGAAGATGCAGAAGCAATGTCTCAAGAGCAAATTGATGAATTAGATAGACTGTCAACAAAACAAGTCCAAGAACACTTTAGTTGGGATTTTATTGTTGATGAGTACGAAGGATTGTTTGGGGAGTTTGATTAA | | | | | | | ATGACTAAATCTGTATACATTATTGGTTCAAAGGGTATTCCAGCTAAATACGGTGGATTTGAAACCTTTGTAGAAAAATTAACTGAAAATCAGAAAAATAAGTCTATCAAATATTTTGTGGCTTGTACTAGAGAGAATTCGCTAAAGTCAAATATTTCGGATGATGTTTTTGAGCATAATGGAGCAACGTGTTTTAGCATTGATGTTCCAAACATTGGTCCAGCAAAGGCTATTGCTTATGATATTGCTGCATTGAAGAAGGCTATTGCTATCTCCAAGCAAAACAAAGATAAGGAACCTATCTTTTATATTTTAGCTTGTCGTATCGGTCCGTTTATCTCAAAATACCGTAAAATCATTCACCAAATGGGGGGGAAACTTTTTGTTAATCCAGATGGTCATGAATGGTTACGAGAAAAATGGAGTGCTCCAGTACGCAGTTATTGGAAACTTTCAGAGTCACTGATGGTAAAATACGCTGACTTATTGATTTGTGATAGTAAAAATATTGAAAAATATATTCAAAATGATTACAGTAAATTTAGTCCTAAAACTACCTATATTGCTTATGGAACTGATTTATCAAAATCTTCTTTAACCTCAAAAGACAGAGTTGTTCGTGAGTGGTTTGATGAAAAGAAAGTTAATGAAAATAGCTATTATTTGGTTGTAGGTCGTTTTGTTCCTGAGAATAACTACGAATCAATGATTCGAGAATTTATGAAGTCAAACTCTAAAAAAGATTTTGTTTTGATTACTAATGTTGAACAAAATGCTTTTTATGAAAAACTCAGAAAAGAAACCGGATTTGATAAAGATAAGCGGATAAAGTTTGTTGGAACAGTCTATAACCAAGAACTTTTGAAATACATTCGTGAAAATGCTTTCGCCTATTTCCATGGTCATGAAGTAGGAGGGACAAATCCATCACTTCTAGAAGCGTTATCATCTACGAAATTAAATCTCCTATTAAATGTTGGCTTTAACAGAGAAGTTGGAGAAGATGGCGCACTATATTGGGATAAAGATGATCTGCACAAGGTTATTGAAGAAAGTGAACAATTGTCACAAGAAAAAATTGATGAAATGGATGAATTATCAACAAAACAAGTCAAAGAAAGATTTTCTTGGAACTTTATTGTTGATGAGTATGAGCAGTTATTTAAAGGATAA | | | | | | | | Sequence (5’to 3’) |
| gBlock of *cpsF* from *S. gallolyticus* GenBank accession FR824043 | | | | | | | gBlock of *cpsF* from *S. suis* GenBank accession AB737838 | | | | | | | | Description |
